# River Plastic Retention and Export across Europe

**DOI:** 10.1021/acs.est.6c03752

**Published:** 2026-10-23

**Authors:** Miranda Stibora, Tim H. M. van Emmerik, Daniel González-Fernández, Albrecht Weerts, Miguel J. Sánchez-Guerrero-Hernández, Gert Everaert, Kryss Waldschläger

**Affiliations:** † Hydrology and Environmental Hydraulics, 4508 Wageningen University and Research, Wageningen 6708 PB, Netherlands; ‡ Department of Biology, 16727 University Marine Research Institute INMAR, University of Cádiz and European University of the Seas, Puerto Real, Cádiz 11519, Spain; § 55955 Deltares, Delft 2629 HV, Netherlands; ∥ Research Department, Flanders Marine Institute, Ostend 8400, Belgium

**Keywords:** Spatially probabilistic model, water quality, marine litter, microplastic, macroplastic

## Abstract

Rivers play a central role in the global distribution of plastic pollution. Despite recent research showing that the majority of plastic is not transported to the sea, most plastic transport models have not explicitly included plastic retention in rivers. We propose a new model to quantify micro- and macroplastic retention on land and in rivers, and export toward the sea. Of the 3.55 Mt year^–1^ of plastics emitted to the environment, only 0.1% of microplastics and 0.5% of macroplastics are exported from the river outlet. This results in 16.9 (15.7–35.1) kt year^–1^ of microplastic and 3.2 (1.4–12.1) kt year^–1^ macroplastic export. Rivers store plastic, retaining 159.0 (147.8–330.6) kt year^–1^ of microplastics (5.3%) and 67.4 (29.7–255.4) kt year^–1^ of macroplastics (2.5%), while the greatest proportion of plastic remains on land. Although large basin size and high plastic emission to land characterize high absolute micro- and macroplastic retention and export, large basin size is a weaker predictor for high plastic retention and export per capita. By aggregating ranks for plastic export and retention, we provide a new and comprehensive outlook on the most polluting rivers of Europe. This work provides crucial baseline estimates for plastic pollution in light of missing plastic reduction quotas to guide future prioritization and assessment of mitigation actions.

## Graphical abstract

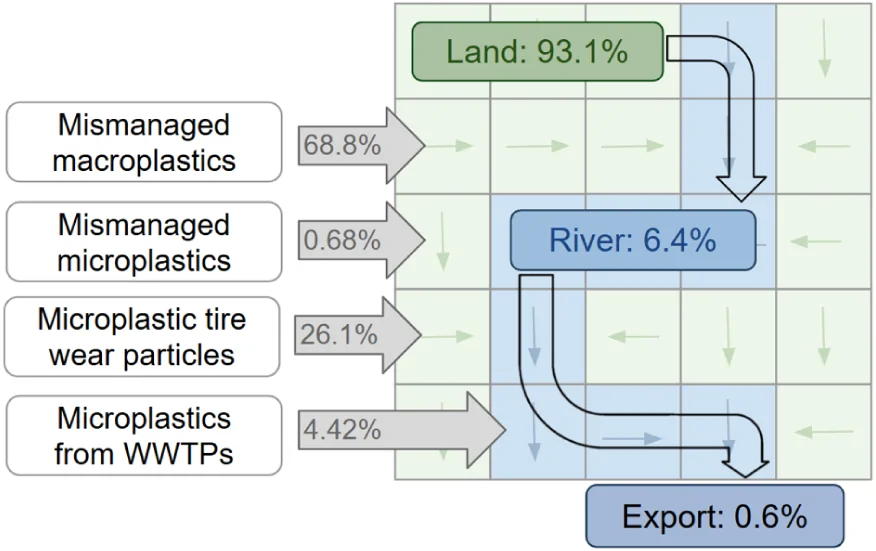

## Introduction

Globally, an estimated 48.3–56.3 million metric tonnes of macroplastic waste was released into the environment in 2020,^1^ and the mass is expected to rise in the coming years,^2^ highlighting the large scale of plastic pollution. Both micro- (<0.5 cm) and macroplastics (>0.5 cm) have been shown to pose significant risks to ecosystems and organisms, with negative effects including entrapment, endocrine disruption, and bioaccumulation in high trophic levels.^3,4^ Rivers play a central role in the transport of plastic pollution, facilitating their movement downstream from their source or retaining them temporarily along the river trajectory.^5,6^ To better understand these processes, rivers are monitored for plastic pollution.^7,8^ Beyond direct monitoring, models can provide insights into the fate of plastics and how they are transported or retained in river basins across wider spatial and temporal scales.

Large-scale modeling of plastic transport can identify rivers that are highly polluted across a large spatial scale. Various large-scale models for plastic transport have been developed, including Lebreton et al.,^9^ Schmidt et al.,^10^ Meijer et al.,^11^ Strokal et al.,^12^ Sonke et al.,^13^ and Nakayama and Osako.^14^ These export models combine population density and mismanaged plastic waste estimates with river basin characteristics to estimate plastic export to the sea. These models only quantify plastic export and overlook the importance of quantifying plastic retention in the river despite recent findings showing that the majority of plastics are likely retained within the river system.^6,11,15^ Another limitation to previous modeling studies is that many models are calibrated with limited data. While macroplastic export models have undergone sufficient calibration by comparing modeled estimates with field measurements (e.g., Meijer et al.,^11^ the steps used to harmonize field data remain under-specified, limiting reproducibility. Microplastic export models remain partially uncalibrated^10,16,17^ due to the complexity and heterogeneity of field measurement protocols. This lack of standardization hinders data harmonization, and authors have reported that extrapolating across inconsistent data sets can lead to the overestimation of microplastic transport.^18^ In light of these challenges, our approach prioritizes thorough data harmonization and model calibration, allowing for a more reliable quantification of plastic retention and export from rivers.

Here, we present a European high-resolution model for micro- and macroplastic export and retention for 8671 river basins. We express our model results in absolute and per capita terms to show the total impact of plastic in river systems and to assess the local leakage risks. We incorporate the latest research in plastic mobilization to explore micro- and macroplastic transport and retention processes across European rivers. Overland transport processes include meteorological drivers^19,20^ and land-use-related mechanisms,^21^ while river transport is shaped by riverbank friction,^22^ hydro-morphological factors^23^ and artificial infrastructures.^24^ We used recent plastic field measurements (i.e., González-Fernández et al.,^8^ Schöneich-Argent et al.,^25^ Piehl et al.,^26^ Pol et al.,^27^ to strengthen the quality in data harmonization for calibration. Our research provides valuable insights into quantifying the proportion of plastic that is retained on land, in the river and exported to the river outlet. This information can be used to provide guidance for designing, implementing, and evaluating plastic pollution reduction measures.

## Methodology

### Probabilistic Model Approach

Our model independently estimates the mass of microplastics (1 μm–5 mm) and macroplastics (>5 mm) that are retained on land, retained within rivers, or exported to the outlet for 8671 European rivers (see Text S1 in Supporting Information) for the year 2015. To estimate this flux, we apply a spatially probabilistic model, developed by building on the framework proposed by Meijer et al.^11^ at a 3 × 3 arc-second resolution (approximately 100 m at the equator). We calculate the likelihood of plastics being transported from mismanaged sources across land, into rivers, and ultimately exported to the river outlet, following flow direction (Figure 1). We define ″export to outlet″ in our model as plastic that is transported to the river outlet boundary, without considering what is transported further to the sea. Estuarine dynamics are not included in this model due to the complexity of tidal and retention processes, a research area still in its early developmental stages.^28,29^

**Figure 1:**
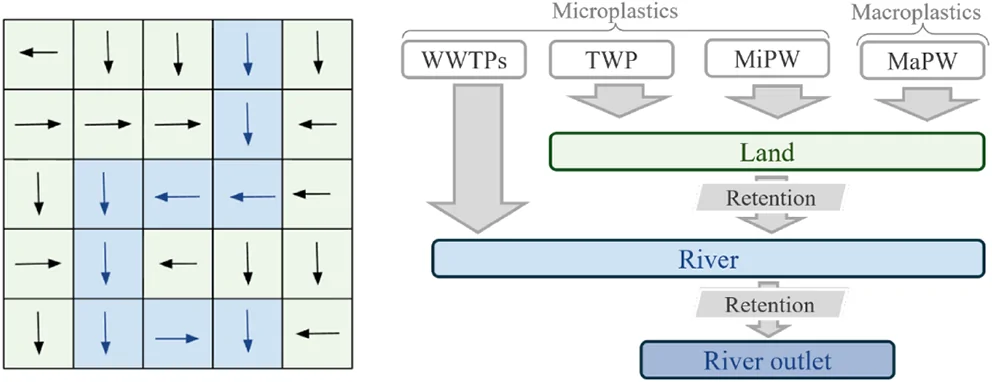
A schematic of the spatially probabilistic model. Land cells are shown in green and river cells in blue, with arrows showing the direction of travel. Three sources of plastic, tire wear particles (TWP) and mismanaged micro- (MiPW) and macroplastic waste (MaPW), are directly introduced onto land, while plastic from wastewater treatment plants (WWTPs) directly enter the river system. All plastic sources that enter the river are then either accumulated along the river trajectory or transported to the outlet of the river system.

### Model Formulation

We determine three key sinks of plastic for a single annual cycle in a closed system: retention on land, retention in the river, and export to the outlet (Figure 1). Transport differences between micro- and macroplastics are accounted for through separate model parametrizations. The model formulation is described in more detail below.

#### Plastic Mobilization on Land

Human activity on land is a key source of plastic.^30^ We account for two key processes, transport due to surface runoff, and wind to quantify plastic transport across land. By estimating the probability (0–1) of plastic mobilization due to wind, *P*(*W*)_*n*_, and surface runoff, *P*(*S*)_*n*_, independently, we determine the fraction of plastic that is mobilized on land, *P*(*L*)_*n*_, at each grid cell_(*n*)_. This is given by

1 $$P(L{)}_{n}=P(W\cup S{)}_{n}=\min((P\left(W{)}_{n}+P\left(S{)}_{n}\right),1\right)$$

The independent processes reflect the experimental setup of Mellink et al.,^20^ which explored the effect of eolian and pluvial processes on plastic mobilization separately. Mellink et al.^20^ showed that larger wind speeds increase the sheer stress that is exerted on micro- and macroplastics, increasing mobilization. Plastic mobilization from wind, *P*(*W*)_*n*_ (0–1), is further affected by the interaction of the driving force of wind, *w*_*n*_ (m s^–1^), and resisting forces, land friction, *lf*_*n*_ ((−)­log_10_ m), and slope, *s*_*n*_ (^◦^), such that

2 $$P(W{)}_{n}=\min\left(\left(c_{w}\cdot w_{n}\right)\cdot \left(c_{lf_{w}}\cdot lf_{n}\right)\cdot \left(c_{s_{w}}\cdot s_{n}\right),1\right)$$

where, the probability of plastic mobilization due to wind for each grid cell_(*n*)_ is between 0 and 1. To quantify land friction, we use the CORINE land cover classes^31^ and apply friction values to each corresponding land class given by Döorenkämper et al.^32^ Friction values from Döorenkämper et al.,^32^ otherwise defined as roughness length (z_0_), used in a wind atlas generation, were applied to the CORINE land classes. Friction values were then transformed using (−)­log_10_ resulting in inverse friction values, with a minimum value of 0.1, to prevent zero mobilization values (see Table S1 in the Supporting Information). The coefficients, denoted by the parameters beginning with *C* (e.g., *c*_*w*_, $c_{lf_{w}}$ , $c_{s_{w}}$ ) are model-derived parameters obtained through model calibration. The values of all coefficients used throughout the following equations are provided in Table S2, in the Supporting Information.

Similarly, we determine that the probability of mobilization by surface runoff is given by the combined effect of the driving force, surface runoff, *sr*_*n*_ (L h^–1^),^33^ and resisting forces, land friction, *lf*_*n*_ ((−)­log_10_ m)^31,32^ and slope, *s*_*n*_ (^◦^). While Meijer et al.^11^ use rainfall as a predictor for plastic mobilization on land, we instead employ surface runoff as an explanatory factor. Research suggests that plastic mobilization can only occur if there is sufficient surface runoff, where positively buoyant plastic can be lifted and mobilized.^19,20^ The combined effect of surface runoff, land friction and slope is given by

3 $$P(S{)}_{n}=\min\left(\left(c_{sr}\cdot sr_{n}\right)\cdot \left(c_{lf_{sr}}\cdot lf_{n}\right)\cdot \left(c_{s_{sr}}\cdot s_{n}\right),1\right)$$

The probability of plastic mobilization due to surface runoff, *P*(*S*), for each grid cell_*(n)*_, is bound within the range 0–1. The same input of land friction and slope is used, however, distinct coefficients are applied to each input ( $c_{lf_{s}}$ and $c_{s_{sr}}$ ) to account for the combined resisting effect with surface runoff. Monthly averaged data for surface runoff and wind speed is used to calculate the average surface runoff and wind speed for the period of 2014–2016,^33^ to match the availability of mismanaged plastic waste.^34^ By accounting for wind, surface runoff, slope, and land friction, we determine the probability of plastic mobilization from one grid cell to the next, *P*(*L*)_*n*_. To find the probability that plastic is transported across land, we find the product of *P*(*L*)_*n*_ for each grid cell along the overland transport pathway to the closest river cell,*P*(*pL*). This is given by

4 $$P(pL{)}_{\left(n,n_{0}\right)}=\overset{n}{\underset{s=n_{0}}{\prod }}P(L{)}_{s}$$

where the transport pathway is the product of the probability of transport on land, *P*(*L*), from the closest river cell, *s* = *n*_0_, to the further in-land cell of interest, *s* = *n*. The transport pathway is given by using flow direction, a digital elevation model (DEM)^35^ and the “Flow Distance” ArcGIS tool. These determine the local drainage direction across land.

#### Plastic Mobilization in the River

Plastic in the river system is affected by the driving force, discharge, and resisting forces, riverbank friction, Strahler order and dams. Discharge is commonly used in models,^11,16^ where an increase in discharge leads to an increase in transport capacity. Strahler order is a proxy for river width given that smaller-order streams with narrower cross sections can experience greater resisting force between riverine plastics and the riverbed.^36^ Cesarini and Scalici^22^ found that the type of riverbank affects the entrapment of plastic along the river trajectory. The same friction values used for land friction^32^ are used for riverbank friction, using the focal average of land friction of land cells surrounding a river cell. Finally, we account for dams,^37^ as these have the potential to trap large quantities of plastic.^38,39^ We calculate the probability that plastic is mobilized in the river, *P*(*R*)_*n*_ (0–1), by

5 $$P(R{)}_{n}=\min\left(p+\left(c_{d}\cdot d_{n}\right)+\left(c_{bf}\cdot bf_{n}\right)+\left(c_{so}\cdot so_{n}\right),1\right)$$

for each river cell, *n*. The first parameter, *p*, serves as an intercept term, indicating the expected probability of plastic transport in the river when contributing factors are zero. The additional additive effect of discharge, *d*_*n*_ (m^3^ s^–1^),^40^ Strahler order, *so*_*n*_, and riverbank friction, *bf*_*n*_ ((−)­log_10_ m), are then capped between 0 and 1. Due to limited knowledge on the proportion of plastic that is entrapped by dams, we cap mobilization at 15% for those river cells.^41^ We determine that the probability that plastic is transported from its river entry to the outlet of the river system, *P*(*pR*), as

6 $$P(pR{)}_{\left(n,n_{0}\right)}=\overset{n}{\underset{s=n_{0}}{\prod }}P(R{)}_{s}$$

where the pathway transport is the product of the probability of riverine transport, *P*(*R*), from the outlet cell, *s* = *n*_0_ to the point of entry in the river, *s* = *n*. This pathway is generated using flow direction and the ArcGIS tool ″Flow Length″. To determine the probability that plastic is transported from land to the river outlet, *P*(*O*), we find the product of the probability of plastic being transported to the river system, *P*(*pL*), and the probability that plastic is transported to the river outlet, *P*(*pR*). This is given by

7 $$P(O{)}_{n}=P(pL\cap pR{)}_{n}=P(pL{)}_{n}\cdot P(pR{)}_{n}$$

for each cell, *n*, in the catchment area, *n*.

#### Fate of Plastic

For each cell in the river basin, *n*, we find the proportion of plastic that is exported to the river outlet by calculating the product of the total emission of plastic, *i* (kg year^–1^), that enters the environment in a cell, *n*, and the probability of plastic export to the river outlet, *P*(*O*). The total quantity of plastic that reaches the outlet of the river basin, *E* (kg year^–1^), is found by aggregating at the individual cells in the river basin, by

8 $$E_{n}=\overset{n}{\underset{s=n_{0}}{\sum }}(P\left(O{)}_{n}\times i_{n}\right)$$

for each basin, *n*. To find the total plastic export, all cells within the river basin are included (from *n*_0_ to *n*). To quantify the total mass of plastic that is retained within the river, *Rr* (kg year^–1^), for each river basin, *n*, at an annual time scale, we calculate:

9 $$Rr_{n}=\left(\overset{n}{\underset{s=n_{0}}{\sum }}(P\left(pL{)}_{n}\times i_{n}\right)\right)-E_{n}$$

First, we calculated the total sum of proportion of plastic that enters the river system, *P*(*pL*) (0–1), from what enters the environment, *i* (kg year^–1^) for all cells (from *n*_0_ to *n*). Second, we found the remainder of the mass of plastic that stays within the river system, by subtracting the mass of plastic that reached the outlet, *E*, for river basin, *n*. The total mass of plastic that is retained on land, *Rl* (kg year^–1^), is given by the following mass balance equation:

10 $$Rl_{n}=I_{n}-E_{n}-Rr_{n}$$

The mass of plastic that is exported, *E* (kg year^–1^) and retained in the river, *Rr* (kg year^–1^), are removed from the total mass of plastic that enters land, *I* (kg year^–1^). The remaining total gives the mass of plastic that is retained on land, for each river basin, *n*.

### Estimating Plastic Emissions to the Environment

To quantify the transport and retention of plastic on land and in rivers, we estimate the mass of micro- and macroplastics that enters the environment as an input to the model. We account for one source of macroplastic, mismanaged macroplastic waste (MaPW, 2.44 Mt year^–1^),^34^ and three sources of microplastic inputs. Three distinct sources of microplastics are defined as a result of their unique emission mechanisms, spatial distributions, and source. We estimate a total mismanagement of 0.02 Mt year^–1^ primary and secondary microplastics emitted to land (MiPW). We further account for tire wear particles as a result of tire abrasion (TWP, 0.93 Mt year^–1^)^42^ and wastewater treatment plants (WWTPs, 0.2 Mt year^–1^), as a point source.^43,44^ A full description of the quantification of the sources of plastic is given in Text S2 and Table S3 in the Supporting Information.

### Calibration and Validation

To calibrate and validate the model, field measurements in 29 rivers for microplastic transport and 36 rivers for macroplastic transport from the existing literature were used. Field measurements, given primarily in count fluxes and mass concentrations, were harmonized and extrapolated to yearly mass fluxes using a methodology from Stibora et al.^45^ (see Table S4 and S5 in the Supporting Information). This methodology comprises six steps for harmonizing microplastic field measurements and seven steps for harmonizing macroplastic field measurements. Using the mean and median concentrations from downstream field measurements, we apply correction factors to account for litter-to-plastic conversion, temporal extrapolation, size-range harmonization, item-to-mass conversion, and vertical extrapolation. A thorough explanation for each harmonization step is outlined in Stibora et al.^45^

A 70%–30% calibration–validation split was used, where rivers were assigned to each group to ensure an even distribution of total plastic emissions and maximum river discharge across both sets. Monte Carlo model runs (around 120 per run) were carried out to calibrate the model, using an initial predefined range of parameter values. Model performance was assessed using *r* ^2^ by comparing modeled yearly plastic flux (log_10_ kg year^–1^) and extrapolated measured yearly plastic flux (log_10_ kg year^–1^). By assessing the individual parameter effect on model output, parameter ranges were refined, and a following seven Monte Carlo model runs were carried out in an iterative process. The remaining field measurements were used to validate the performance of the best model run, which resulted in the highest *r* ^2^ from model calibration.

Following model calibration and validation, confidence intervals were estimated. Using the best performing model run, we performed a sensitivity analysis by adjusting all model coefficients by ± 10% in a one-at-a-time (OAT) framework. 20,000 model runs were carried out for six basins representing the median basin size. Confidence intervals were then estimated using the 10th to 90th percentile range of the resulting model results. Results from the OAT can be found in Table S6 and S7 in the Supporting Information.

### Data Analysis

We express micro- and macroplastic retention and export in terms of absolute and normalized values for each river basin in Europe for 2015. We normalized retention and export estimates using population density data from 2015.^46^ To assess the characteristics of the most polluting rivers in terms of absolute and per capita estimates for river retention and export, we used a subset of the top 1% (*n* = 87) of rivers for each plastic fate. We further express the median export and retention (absolute and per capita) for each group of basin size classes, < 10, 10–100, 100–1000, and >1000 km^2^. To assess the overall level of pollution of a river basin, we assigned and aggregated river basin ranks for each of the four fates of plastic, micro- and macroplastic retention, and export. Spearman rank tests were also carried out to find the statistical relationship between the two groups of ranks. We compare micro- and macroplastic export estimates for the largest 15 rivers of Europe to other large-scale plastic export models. Macroplastic export estimates were compared to estimates from Lebreton et al.,^9^ Schmidt et al.,^10^ Meijer et al.,^11^ and Strokal et al.,^16^ and microplastic export estimates were compared to those from Schmidt et al.,^10^ Strokal et al.,^16^ Siegfried et al.,^17^ and Weiss et al.^18,47^

## Results and Discussion

### Plastic Export Model Performance

From model calibration, we find that our results for micro- and macroplastic transport corresponded well with field data, resulting in *r* ^2^ values of 0.61 for microplastics and 0.79 for macroplastics (Figure 2). Results of the model validation confirmed that the model was also able to predict the plastic transport in rivers that were not used in the calibration set (0.88 for microplastic transport and 0.77 for macroplastic transport).

**Figure 2:**
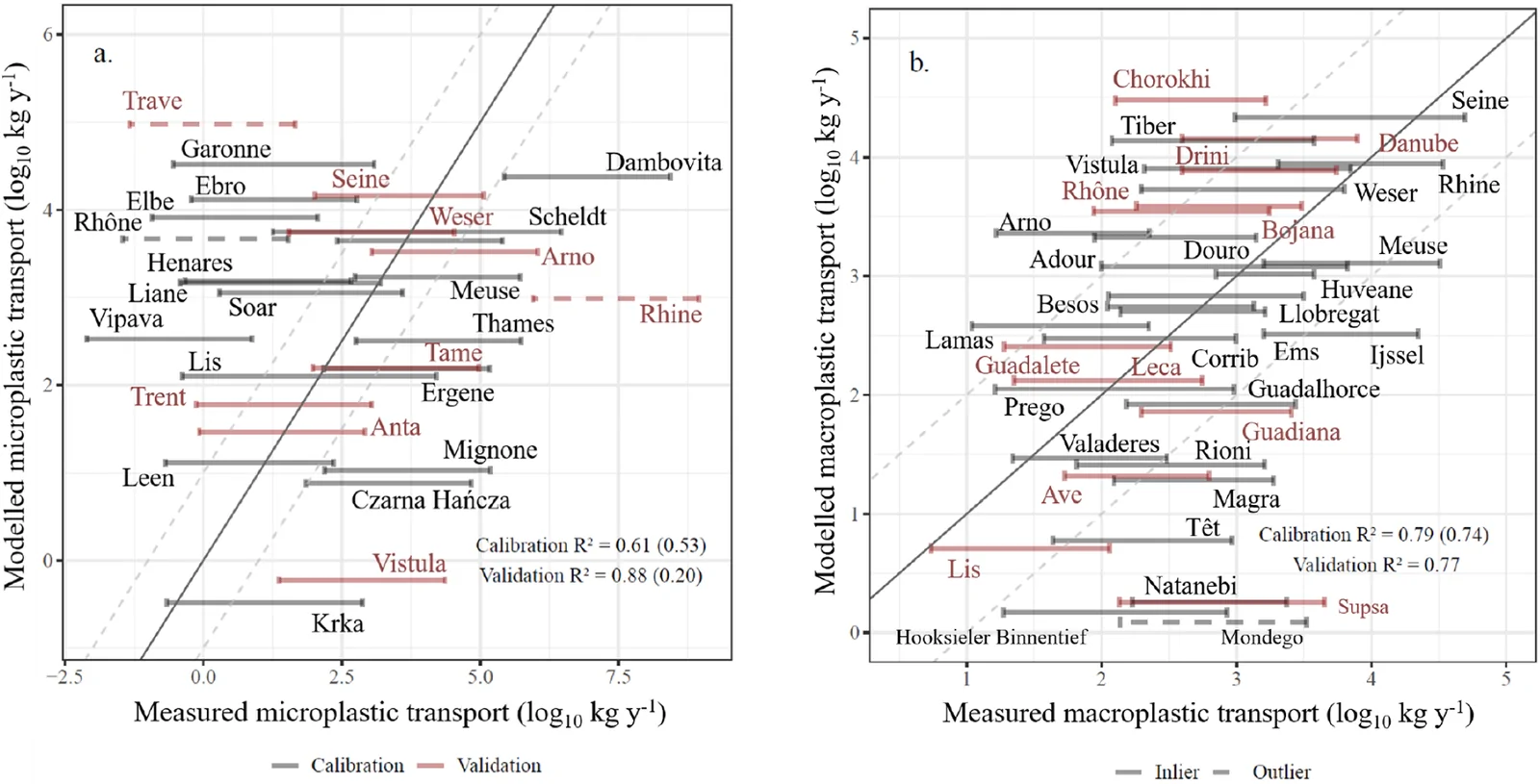
Results from the model calibration and validation for (a) micro- and (b) macroplastic transport compare the modeled yearly flux with the range in extrapolated field measurements. Rivers in the calibration set are given in black and validation in red. Model performance estimates (*r* ^2^) are shown for the best performing model run for calibration and validation rivers. Performance estimates in brackets show *r* ^2^ including outliers, while estimates outside the brackets have the outliers removed. A 1:1 line is also included, gray dashed lines to show 1 order of magnitude difference.

As a result of field measurement extrapolation and limitations in the model, several rivers were identified as outliers and were removed from the model performance analysis. We identified 3% (n = 1) of rivers as being outliers for macroplastic transport and 10% (n = 3) rivers for microplastic transport. These modeled values differed from the extrapolated field values by more than 2 orders of magnitude. While we tried to identify field-methodological factors (sample volume and mesh size) to explain the outlying rivers, we found no clear differences compared with nonoutlying rivers that exhibited the same methodological limitations. For the calibration set, we found that, with the inclusion of outliers, the model performance for macroplastic export (*r* ^2^ of 0.75) was higher than that for microplastic export (*r* ^2^ of 0.53). While field measurement extrapolation uncertainty stems from field technique limitations (e.g., limited size range coverage and duration), model uncertainty stems from our incomplete understanding of the sources of plastics in the environment, transport and retention processes, and model formulation. These uncertainties highlight the importance of comparing model results to field measurements and ensuring transparency in estimating plastic transport. Overall, however, the model calibration demonstrates a strong performance, providing confidence in its ability to capture the most relevant transport dynamics.

### Less Plastic Is Transported to the River Outlet Than Previous Estimates

We estimate that a very small proportion of plastic that is released to the environment is transported to the river outlets (Figure 3). Of the 3.55 Mt year^–1^ of plastic that is released into the environment in Europe, only 17.1–47.2 kt year^–1^ (0.14% of all microplastics and 0.45% of all macroplastics) is exported to the outlets of all rivers (Figure 3a). The proportion of plastic that is transported to the river outlet deviates from the research work of Meijer et al.^11^ Meijer et al.^11^ stated that globally, on average, 2% of all mismanaged macroplastic is exported to the river outlet. This deviation is consistent with broader research on riverine plastic export, which has highlighted the potential overestimation of plastic fluxes to the sea in previous estimates by 2 to 3 orders of magnitude.^18^

**Figure 3:**
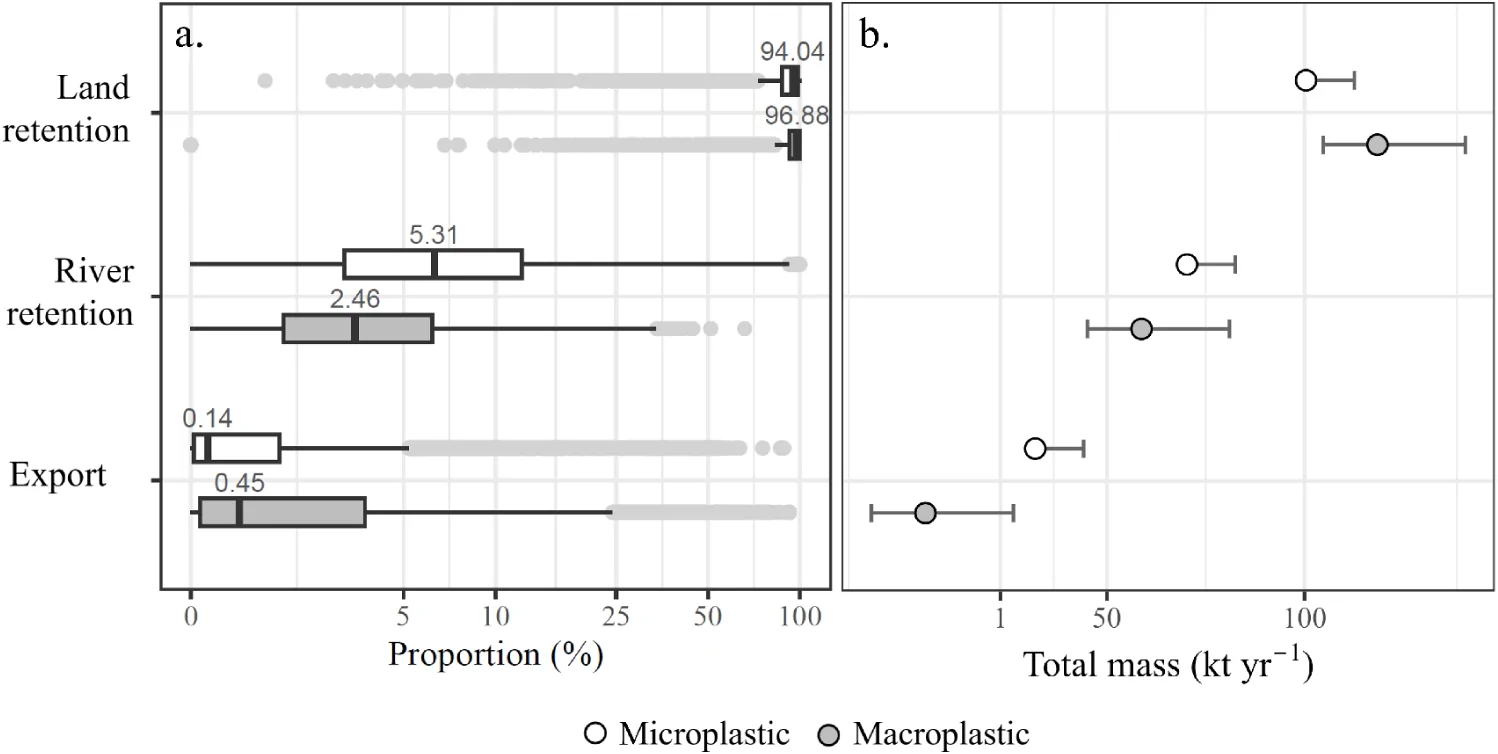
(a) Range in the proportion (%) of micro- and macroplastic that is retained on land, retained in the river system, and exported to the river outlet. (b) We present the total mass of plastic export and retention in river and land for all European basins. The total export of micro- and macroplastics are 16.9 (15.7–35.1) and 3.20 (1.4–12.1) kt year^–1^, with river retention totaling 159.0 (147.8–330.6) and 67.4 (29.7–255.4) kt year^–1^, respectively. The total quantity of macroplastic retention on land equates to 2373.7 (1044.4–8996.3) kt year^–1^, while total microplastic retention on land is 933.0 (867.7–1940.6) kt year^–1^.

Estimating the total mass of plastic that is transported to the river outlets of the four main seas that border Europe (North-East Atlantic region and the Mediterranean, Baltic, and Black seas), we find that the North-East Atlantic receives the highest mass of plastic waste from rivers (9.08 kt year^–1^). The Mediterranean Sea follows, with an export of 6.90 kt year^–1^. The Baltic Sea and Black Sea are estimated to export 3.20 kt year^–1^ and 0.91 kt year^–1^, respectively.

Results from the model show that rivers are an important sink for plastics. We estimate that 9 times more microplastics (159.0 kt year^–1^), and 21 times more macroplastics (67.4 kt year^–1^) are retained in all river systems compared to the total mass that is exported for each plastic size category (Figure 3b). These findings are also supported by van Emmerik et al.,^6^ which state that rivers are important for the retention of plastic. The final and largest proportion of plastic is retained on land (94.0% of all microplastics and 96.9% of all macroplastics). This results in a total retention of 0.9–1.9 Mt year^–1^ of microplastics and 1.0–9.0 Mt year^–1^ macroplastics on land (Figure 3b). Model results show a large inter-basin variability in Figure 3a, where the proportion of plastic export and river retention ranges from 0–100% across basins. Basins with the highest proportion of micro- and macroplastic export (>25%) in Figure 3a are characterized by a small basin size, with a large share of plastic emissions to the environment entering the basin close to the river outlet. Further understanding the characteristics of rivers that result in either a high or low retention and export can offer us useful insights into understanding the overall pollution levels of a river system.

### Comparing Transport-to-Outlet Estimates with Other Model Estimates

Modeled export estimates vary greatly between models (Figure 4), with estimates differing by as much as 2–4 orders of magnitude. We find that the majority of our main river estimates (80% of macroplastic and microplastic export estimates) fall within the minimum and maximum of the other results. In addition to variations in model setup, variations in model estimates stem from differences in quantifying plastic emissions and model calibration. Our model, Meijer et al.,^11^ and Strokal et al.^16^ use estimates from Lebreton and Andrady^34^ for MaPW, while Lebreton et al.^9^ and Siegfried et al.^17^ use different estimates. For microplastic export, our model integrates three microplastic sources: MiPW, TWP, and WWTPs. Schmidt et al.^10^ account solely for MiPW, while Strokal et al.^16^ account for WWTPs and MiPW, and Siegfried et al.^17^ account solely for point sources (WWTPs). Weiss et al.^18^ and Weiss et al.^47^ do not account for specific sources when modeling microplastic flux. Instead, Weiss et al.^18^ estimate microplastic flux to the sea using field measurements, while Weiss et al.^47^ use these microplastic flux estimates to model the fate of microplastics at sea using Lagrangian modeling.

**Figure 4:**
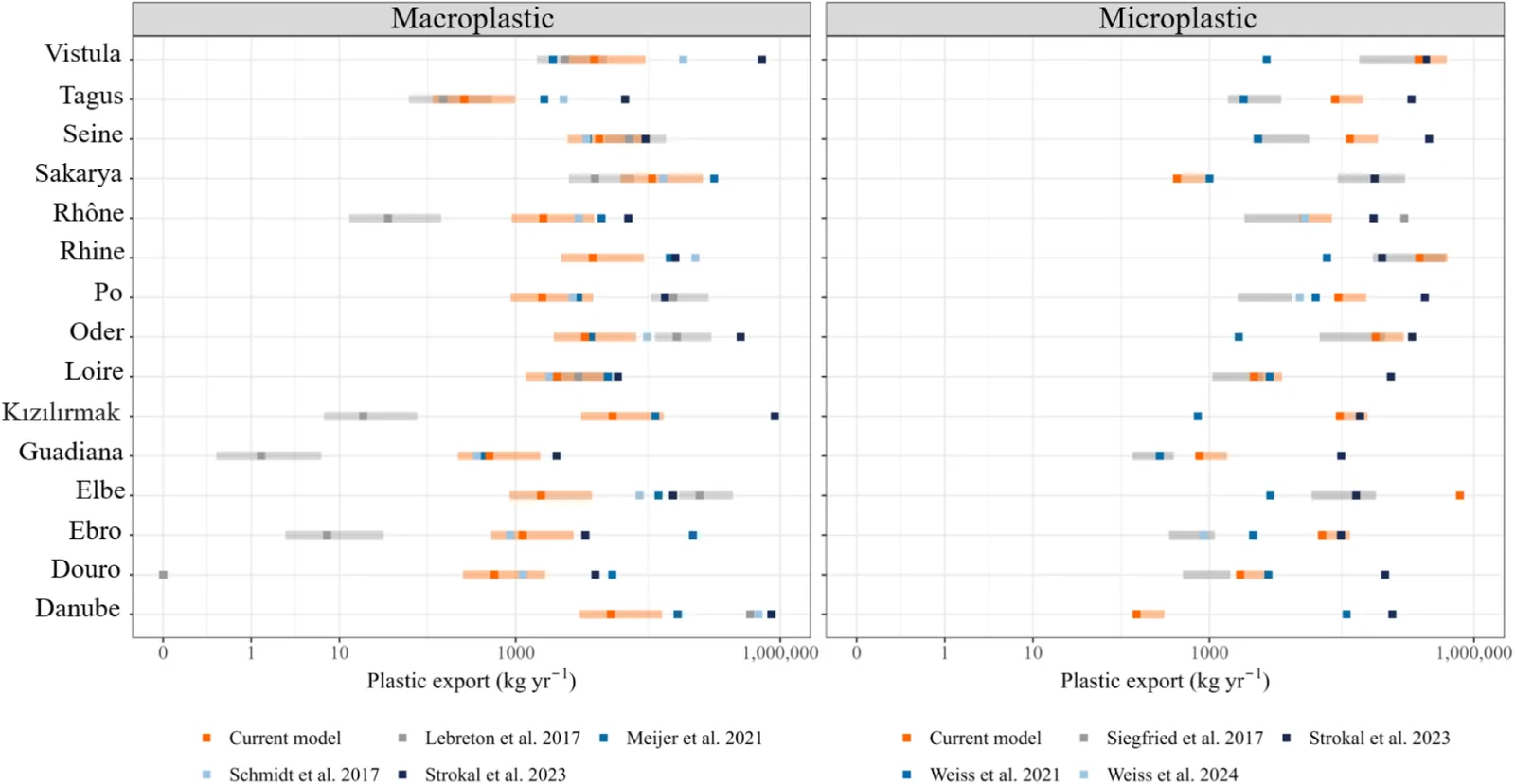
Range in micro- and macroplastic export values from various large-scale plastic transport models for the 15 largest rivers of Europe. Different colors denote different models, with separate color scales used for micro- and macroplastic export estimates. The same color categorization is used for papers where both micro- and macroplastic export are estimated.

Regarding calibration, our model and Meijer et al.^11^ use field data for the Rhine for macroplastic model calibration.^23^ While Meijer et al.^11^ extrapolated field measurements, estimating an observation-based yearly plastic transport of 38 t year^–1^, we estimate 2–34 t year^–1^. Prior modeling efforts for microplastic export have lacked the extensive calibration methodology implemented here due to prior reduced data availability.^9,10,16,17^ While the large heterogeneity in sampling protocols used to estimate microplastic flux (e.g., size range measured and characterization methodology) increases the uncertainty of extrapolating microplastic concentration measurements, applying a consistent and reproducible methodology to harmonize field samples allows us to provide a transparent estimate of microplastic flux suitable for model calibration.^45^ Transparency in quantifying plastic sources and data handling for model calibration are, therefore, crucial when comparing model estimates.

We find that models with a higher resolution are better able to account for the majority of plastic export, compared to low-resolution models. We estimate that 239 (2.8%) river systems contribute 80% of macroplastic export, while 141 (1.6%) rivers account for 80% of microplastic export. Selecting only European rivers, Meijer et al.^11^ estimated that 188 (7.4%) rivers contribute to 80% of plastic waste at the river outlets, while Lebreton et al.^9^ estimated 18 (0.4%), and Schmidt et al.^10^ only 11 (0.8%). Coarser models^9,10^ may underestimate or fail to model plastic transport in small river basins, which can have a substantial impact on total plastic export due to their proximity to the coastal region.^48^ Accurately estimating the mass of plastic reaching river outlets requires accounting for the flux contributed by small drainage basins, which requires a model with high resolution.

### High Absolute Export and Retention Estimates Are Associated with Large Basin Size and Large Plastic Emissions to the Environment

High absolute estimates for plastic fate (top 1%) are strongly associated with large basin areas (Figure 5). This relationship is particularly pronounced for high retention estimates, with the median basin size being 115 and 399 times greater than the median basin size for macro- and microplastic retention. These results are supported in Table 1 where the largest size class (>1000 km^2^) consistently shows the highest estimates across all absolute estimates for micro- and macroplastic retention and export. Schmidt et al.^10^ also report that large river basins receiving substantial plastic emissions result in a large plastic export. Despite the association of large basin size with high absolute estimates, rivers with the 20 highest estimates for macroplastic export are medium coastal basins (80 km^2^) (Figure 5). These are only three times greater than the median basin size for all basins. This is consistent with Lebreton et al.^9^ and González-Fernández et al.,^8^ who show that coastal basins often have high export efficiency. These coastal regions are characterized by intense plastic emissions to the environment, enabling even smaller basins to become disproportionately large macroplastic exporters.

**Figure 5:**
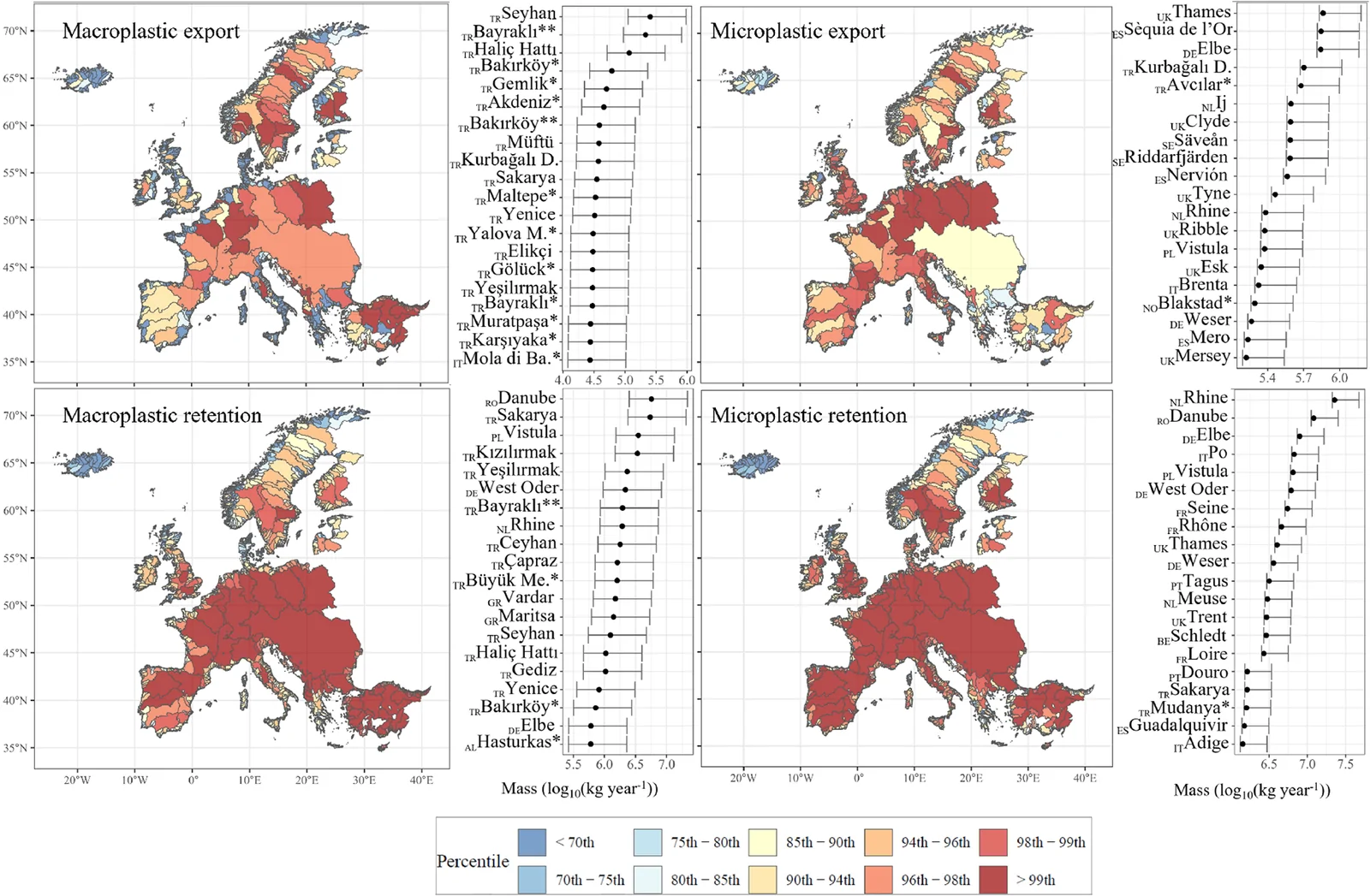
Spatial distribution of rivers with various levels of absolute plastic export and retention. Colors represent percentile classes of estimated plastic export and retention, with darker red shades indicating higher percentiles. Percentile classes are given in the (SI Table S8). Individual absolute estimates for the 20 highest rivers are also displayed.

**Table 1:** Median Absolute and Per Capita Estimate for Micro- and Macroplastic Export and Retention Based on Basin Size Groups

Basin size	Macroplastic exp.		Microplastic exp.		Macroplastic ret.		Microplastic ret.	
(km^2^)	(kg)	(kg cap^–1^)	(kg)	(kg cap^–1^)	(kg)	(kg cap^–1^)	(kg)	(kg cap^–1^)
<10	0.8	0.16	2.5	0.6	0.5	0.08	16.2	4.0
10–100	5.0	0.3	1.7	0.1	17.5	0.9	64.1	5.0
100–1000	12.2	0.09	25.9	0.3	329	1.7	970	6.6
>1000	90.7	0.03	2362	1.3	5349	2.0	25372	11.0

High absolute micro- and macroplastic retention and export (top 1%) are also characterized with large plastic entry to the environment. The median emission of plastic to the environment is more than 1,174 and 6,170 times greater for high plastic retention for micro- and macroplastics, while the median emission is more than 267 and 1,639 times greater for micro- and macroplastic export compared to the median emission to land at the basin level. Results from Strokal et al.^12^ and Meijer et al.,^11^ also reflect these results, with basins characterized by large emissions of plastic from dense urbanization showing high plastic export. While we provide estimates for plastic export and retention, we note that our model currently does not account for floodplain connectivity, which may result in the over- or underestimation of plastic retention on rivers after large flooding events.^49^ Recent research by Schreyers et al.^49^ has shown that such events can greatly increase riverbank retention of plastic, highlighting this as an important transport mechanism for future model incorporation. Despite this, our model provides a good indication of the overall total export and retention patterns. These absolute patterns are vital in showing the total impact of plastic on the environment, which can be used to guide regional and national mitigation strategies and policies. However, these estimates often disproportionately show the effects of extensively populated areas. By accounting for population density, it is possible to explore plastic pollution patterns across river basins, by isolating population intensity from basin size. This enables a more equitable comparison of river basins by balancing differences in population distribution and hydrological basin characteristics rather than attributing plastic pollution to solely based on absolute emissions.

### High Per Capita Estimates Are Associated with a High Quantity of Mismanaged Plastic

A large basin size is no longer a reliable predictor of high plastic export and retention when scaled by population density. For rivers with high plastic retention and export, the median surface area is comparable to the median basin size of all modeled basins (19.4–44.2 km^2^ compared to a median basin size of 29.5 km^2^). Smaller basins tend to have a lower discharge, which reduces the driving force of riverine transport, promoting plastic retention.^50^ Small coastal basins also often have large populations close to the river outlet, which can promote a higher export efficiency,^8^ in addition to WWTPs promoting the direct transport of plastic to the river outlet. Despite this, when accounting for all river basins, we find that the largest basin size group (greater than 1000 km^2^) has the highest estimates for microplastic export and retention, and macroplastic retention (Table 1). We find that plastic pollution scaled by population density, therefore highly depends on the context of mismanaged plastics.

Large emissions of plastic to the environment strongly drive high microplastic retention and macroplastic retention and export scaled by population density, while the locations of WWTPs play an important role in elevated estimates for microplastic export. High macro- and microplastic retention (>96^*th*^ percentile) is found primarily in southeast Europe, while basins with high microplastic retention (85–96^*th*^ percentile) are found in main, south, and northern Europe (Figure 6). These variations are linked to sources of plastic, with MaPW emissions dominating southeastern Europe^34^ and WWTP density dominating main, south, and northern Europe.^44^ Surprisingly, basins with high microplastic export contribute only 1/7 of the total microplastic emissions to land compared to all basins. These rivers have a single large point source (WWTP) close to the river outlet and small population densities upstream, which disproportionately affect per capita estimates. Since per capita estimates are more suited to informing large-scale policy decisions, careful data interpretation is required to ensure that these distorted predictions are accounted for.

**Figure 6:**
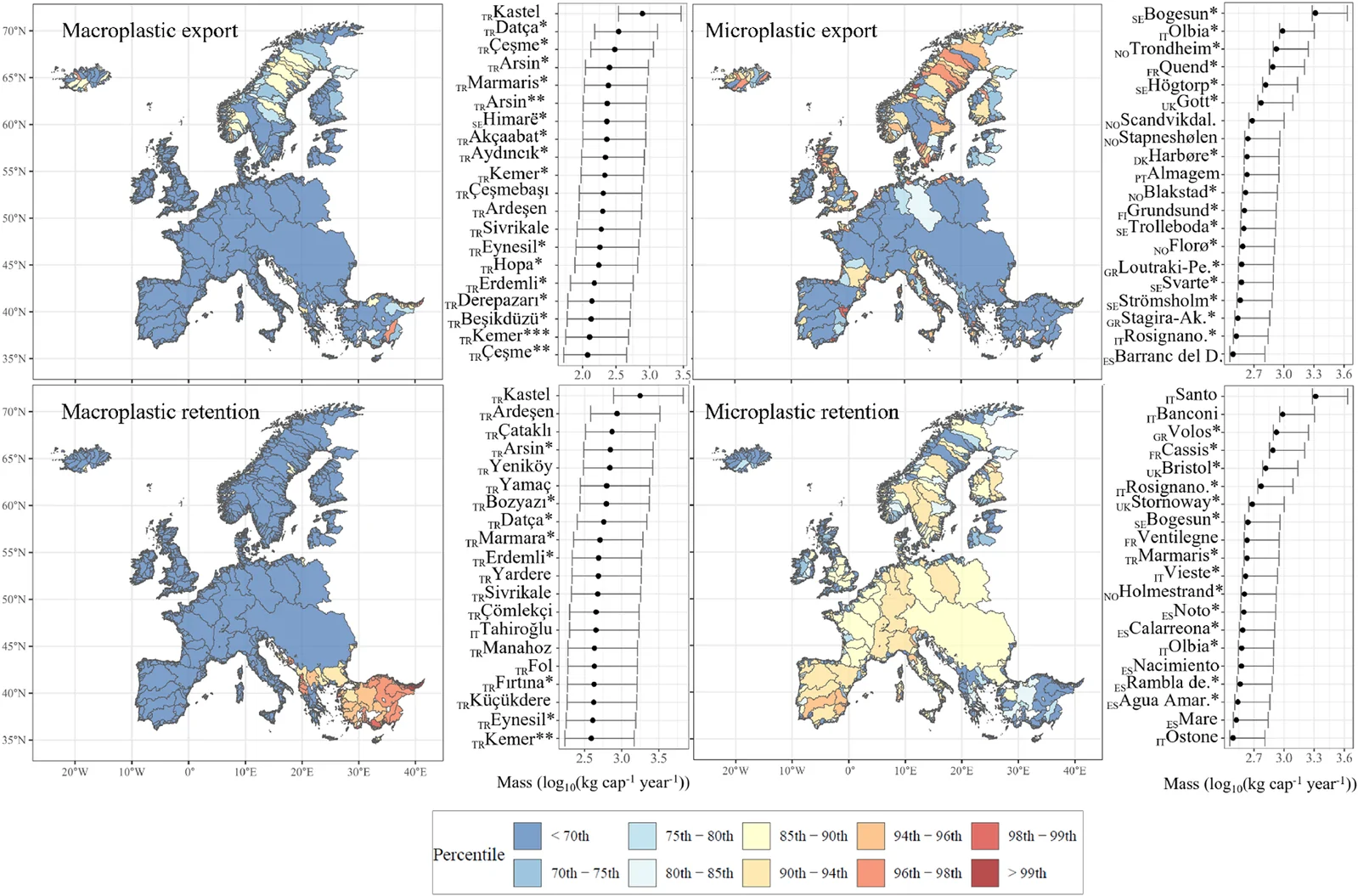
Spatial distribution of rivers with various levels of plastic export and retention scaled by total population density. Percentile classes of a darker shade indicate higher percentile classes. These classes are given in the (SI Table S9). Individual estimates for the 20 highest export and retention scaled by population density are shown.

### Aggregating Plastic Fate Rankings Provides a New Look on River Basin Pollution

By applying an overall rank to each river system, we find that the most polluting rivers are Kurbagalı Dere, Vistula, and Rhine (Table 2). We observe a large range in individual ranks for absolute micro- and macroplastic export and river retention (1–130). The river with the highest macroplastic export (Seyhan) and retention (Danube), and the highest microplastic export (Thames) fall outside the top 10 most polluted rivers. With the quantification of plastic retention being overlooked in plastic transport models,^11,16^ we show that accounting for retention is very important when assessing the overall level of plastic pollution. Plastic export alone provides a skewed perception of riverine plastic pollution. The Vistula, Rhine, and West Oder have higher retention ranks than their export ranks. These combined ranks can be used to identify the total environmental burden that each river system has.

**Table 2:** Overall Rank of the Highest 10 Ranked Rivers for Micro- and Macroplastic Export and Retention Are Displayed, Including Their Fate-Specific Ranks^a^

	Export			River retention	
River	Overall	Macroplastic	Microplastic	Macroplastic	Microplastic
Kurbagalı Dere (TR)	**1**	9	4	24	43
Vistula (PL)	**2**	79	14	3	5
Rhine (NL)	**3**	82	12	8	1
Mälaren (SE)	**4**	32	9	65	34
West Oder (DE)	**5**	94	44	6	6
Tiber (IT)	**6**	36	40	69	23
Weser (DE)	**7**	107	18	39	10
Kızılırmak (TR)	**8**	41	97	4	47
Seine (FR)	**9**	70	81	32	7
Garonne (FR)	**10**	130	21	86	24
Thames (UK)	**18**	410	1	46	9
Seyhan (TR)	**26**	1	498	14	65
Danube (UA)	**72**	44	1116	1	2

a The Thames, Seyhan, and Danube are also shown , as they have the highest rank for a plastic fate.

Assessing the correlation between pairs of export and retention ranks for plastic pollution, we find a positive relationship for each pair of ranks (0.48–0.79) (see Table S10 in the Supporting Information). The strongest positive correlation, with the highest *rho* value, was observed between macroplastic export rank and macroplastic river retention rank (0.793). This indicates that rivers with high macroplastic export also tend to have high macroplastic retention. Since all Spearman’s rank pairs showed positive relationships, this further suggests that a river with a high macroplastic export rank is also likely to exhibit a high microplastic export rank, as well as high retention ranks for both macro- and microplastics. While this positive correlation implies that information on one plastic fate has the potential to show the level of pollution of other fates, it does not imply redundancy when assessing the final rank of a river. This is highlighted by the fact that none of the observed *rho* values reached unity, suggesting that plastic sources, river-specific characteristics, and hydrometeorological variables influence export and retention dynamics for microplastics and macroplastics. Strokal et al.^16^ state the same river system can differ greatly in micro- and macroplastic export. This is shown clearly in Table 2 for the River Thames, which has a high ranking for microplastic export (1), yet a lower ranking for macroplastic export (410). This difference shows that while a high macroplastic export may often be paired with a high microplastic export, variability between river systems requires a combined assessment.

### Limitations

For our model estimates, we use 2015 as the reference year to assess the proportion of plastic retained on land, retained in rivers, and transported to the river outlet. This choice aligns with the global data sets on mismanaged plastic waste^34^ and wastewater treatment plants^44^ used in this study. As this reference year is also widely used across the macroplastic modeling literature,^11,16^ it enhances comparability with existing large-scale models. Furthermore, we note that the majority (75%) of calibration and validation measurements were collected between 2015 and 2018, closely clustered around our chosen reference year. We found that our estimates largely fall within the range of other large-scale models, which builds confidence in the additional novel retention component of our model. While our main goal was to identify the role of plastic retention in large-scale budget estimates and to assess the overall pollution level of river systems, we acknowledge that total plastic emissions to land have increased since 2015.^2^ Despite this increase in emissions, we expect our findings regarding the relative fate of plastics to remain stable. By making our model and input sources openly available, we facilitate updating the framework with more recent data.

Another limitation of our model is that it provides an estimate of plastic retention and export for a single year and therefore, does not account for year-to-year accumulation. This is due to the fact that the current model formulation does not resolve where plastic is spatially accumulated on land or in the river at the end of the run. Future work should focus on extending the model formulation to include an end-of-year spatial distribution to start the following year’s run. Such a development would also facilitate the additional implementation of mitigation strategies, such as clean-ups, to reduce the risk of overestimating long-term accumulation. To verify the model’s ability to estimate multiyear plastic transport, recalibration using multiyear data sets would greatly enhance model confidence.

### Summary and Future Outlook

Our research shows that rivers are important storages for micro- and macroplastics, with our model estimating a reduced plastic export compared to other models for our 2015 reference year. In comparing model results with similar large-scale export studies, we find that modeling plastic fluxes at large scales requires a high model resolution, as small river basins contribute a large proportion to the European plastic retention and export. While absolute estimates provide a good indication of the overall environmental burden and may profit regional management, per capita estimates for plastic export and retention facilitate policy development by allowing for a fair comparison between river basins. This also highlights the importance of the choice in metrics. Furthermore, by combining export and retention scores for micro- and macroplastics to give a final score, we are able to provide a more holistic overview of the overall level of pollution of a river system. We finally emphasize the requirement of future modeling work for implementing extreme events (e.g., floods) and tidal dynamics. Both processes have the potential to significantly affect the transport and retention of plastic in rivers.

## Supplementary Material



## Data Availability

The full data set and model code are publicly available on Zenodo (DOI: 10.5281/zenodo.23055069).

## Notes

Text S1. River basin delineation. Table S1. A harmonization table for CLC and GLC2000 land-use classes. Table S2. Coefficient values for micro- and macroplastic transport on land and in rivers. Text S2. Estimating plastic emissions to land. Table S3. The total mass of each plastic source for all modeled river basins in Europe. Text S3. Calibration and Validation: Harmonised field data. Table S4. Extrapolated field macroplastic transport measurements. Table S5. Extrapolated field microplastic transport measurements. Table S6. Results from the OAT analysis used to calculate the uncertainty of macroplastic export. Table S7. Results from the OAT analysis used to calculate the uncertainty of microplastic export. Table S8. Absolute percentile classes used in Figure 5 in the main text; Table S9. Per capita percentile classes used in Figure 6 in the main text. Table S10. Results from the Spearman’s rank coefficient tests between micro- and macroplastic export and retention ranks. The model setup and calculations are primarily carried out in R software (version 4.4.1). In addition, several geoprocessing steps are performed using ArcGIS and QGIS. All input sources, results, and code have been made open-source to promote open and FAIR science principles (PDF)
