# Supplementary material for: River Plastic Retention and Export across Europe
Source: Environ Sci Technol. 2026 Oct 23;60(44):31944–54. doi: 10.1021/acs.est.6c03752 (PMC13757957; doi:10.1021/acs.est.6c03752)
Supplement: Supplementary file 1 [file es6c03752_si_001.pdf]

River plastic retention and export across Europe

Miranda Stibora,[*,†] Tim H. M. van Emmerik,[†] Daniel González-Fernández,[‡] Albrecht Weerts,[¶] Miguel J. Sánchez-Guerrero-Hernández,[‡] Gert Everaert,[§] and Kryss Waldschläger[†]

†*Hydrology and Environmental Hydraulics, Wageningen University and Research, Wageningen, Netherlands*

‡*Department of Biology, University Marine Research Institute INMAR, University of Cádiz and European University of the Seas, Puerto Real, Cádiz 11519, Spain*

¶*Deltares, Delft, Netherlands*

§*Research Department, Flanders Marine Institute, Ostend, Belgium*

E-mail: miranda.stibora@wur.nl

Contents

Text S1. River basin delineation . . . 3

Table S1. Part 1. A harmonisation table for CLC and GLC2000 land-use classes . . . 4

Table S1. Part 2. A harmonisation table for CLC and GLC2000 land-use classes . . . 5

Table S2. Coefficient values for micro- and macroplastics transport on land and in rivers 6

Text S2. Estimating plastic emission to the environment . . . 7

Table S3. The total mass of each plastic source for all modelled river basins in Europe 7

Text S3. Calibration and Validation: Harmonised field data . . . 10

Table S4. Part 1. Extrapolated field macroplastic transport measurements . . . 10

Table S4. Part 2. Extrapolated field macroplastic transport measurements . . . 11

Table S5. Extrapolated field microplastic transport measurements 12
Table S6. Results from the OAT analysis used to calculate uncertainty of macroplastic export. 13
Table S7. Results from the OAT analysis used to calculate uncertainty of microplastic export. 13
Table S8. Absolute percentile classes, used in Figure 5 in the main text 14
Table S9. Per capita percentile classes, used in Figure 6 in the main text 14
Table S10. Results from the Spearman's rank coefficient tests, between micro- and macroplastic export and retention ranks 15
References 16

Text S1. River basin delineation

To delineate river basins within Europe, a digital elevation model (DEM) of 3 x 3 arc seconds was used.[1] For countries found primarily above 60 degrees latitude (Sweden, Norway, Faroe Islands, Greenland, Iceland, and Finland) a 15 x 15 arc second DEM was used.[1] This was due to the unavailability of a higher resolution HydroSHEDS DEM at this latitude. Based on a visual observation of the spatial overlap between river networks[2] and calculated flow accumulation, we determined a river cell as having an upstream drainage area of roughly 11 km^2 (1400 cells). For high latitude regions with a lower resolution, we assumed an upstream flow area to be around 22 km^2 (50 cells).

Using flow direction and the river outlet point (selected cell with the largest flow accumulation in a river basin), we delineated all drainage basins in Europe. Basins which did not have their outlet at the ocean (>100) were removed from the model. River basins whose area was not fully covered by the Coordination of Information on the Environment (CORINE) land cover database,[3] were also removed from the model set with the exception of the Danube and Vistula river. The Danube and Vistula remained in the model set due to their significance as major European rivers, and availability of field measurement data used for model calibration and validation. Global land cover (GLC200)[4] data was used where CORINE was not available (see Subsection: Retention on land). River basin delineation resulted in the identification of 10861 unique river basins. River basins with a total population density smaller than one were removed from the set ($n = 2190$). This results in 8671 unique river basins.

Table S1. Part 1. A harmonisation table for CLC and GLC2000 land-use classes. The original fraction values from Dörenkämper et al.[5] are presented in addition to the inverse logarithmic transformation used as the friction factor for plastic transport. A minimum friction factor of 0.1 is applied to avoid multiplication by zero when calculating plastic transport

CLC (class number)	GLC2000 (class number)	Friction factor (original value, m)
Continuous urban fabric (111), Discontinuous urban fabric (112)	Artificial surfaces & associated areas (22)	0.1 (1)
Broad-leaved forest (311), Coniferous forest (312)	Tree cover, broadleaved, evergreen (1), Tree cover, broadleaved, deciduous, closed (2), Tree Cover, needle-leaved, evergreen (4), Tree Cover, needle leaved, deciduous (5)	0.1 (0.9)
Industrial or commercial units (121)	-	0.15 (0.7)
Port areas (123), Airports (124), Mineral extraction cites (131), Dump sites (132), Construction sites (133), Green urban areas (141), Sport and leisure facilities (142) Mixed forest (313)	Tree Cover, mixed leaf type (6)	0.30 (0.5)
Road and rail networks (122), Vineyards (221), Fruit trees and berry plantations (222), Olive groves (223), Annual crops associated with permanent crops (241) Complex cultivation patterns (242) Land principally occupied by agriculture (243) Agro-forestry areas (244)	Cultivated and managed areas (16), Mosaic: Cropland / Tree cover (17), Mosaic: Cropland / Shrub or Grass Cover (18)	0.70 (0.2)
Moors and heathland (322), Sclerophyllous vegetation (323), Transitional woodland-shrub (324)	Tree cover, broadleaved, deciduous, open (3), Mosaic: tree cover other nat. vegetation (9), Shrub cover, closed-open, evergreen (11) Herbaceous Cover, closed-open (13) Shrub cover, closed-open, deciduous (12)	0.92 (0.12)

Table S1. Part 2. A harmonisation table for CLC and GLC2000 land-use classes. The original fraction values from Dörenkämper et al.[5] are presented in addition to the inverse logarithmic transformation used as the friction factor for plastic transport. A minimum friction factor of 0.1 is applied to avoid multiplication by zero when calculating plastic transport

CLC (class number)	GLC2000 (class number)	Friction factor (original value, m)
Beach dunes sands (331), Bare rocks (332), Sparsely vegetated areas (333), Burnt areas (334)	Tree cover, burnt (10), Sparse herbaceous or sparse shrub cover (14), Bare areas (19)	2 (0.01)
Glaciers and perpetual snow (335), Inland marshes (411), Peat bogs (412), Salt marshes (421), Salines (422), Intertidal flats (423)	Tree Cover, regularly flooded, fresh water (7), Tree Cover regularly flooded, saline water (8), Regularly flooded Shrub / Herbaceous Cover (15), Snow and Ice (21)	3 (0.001)
Water courses (511), Water bodies (512), Coastal lagoons (521) Estuaries (522), Sea and ocean (523)	Water Bodies (20)	4 (0.0001)

Table S2. Coefficient values for micro- and macroplastics transport on land and in rivers. Values represent unitless coefficients.

Coefficient value	Macroplastic	Microplastic
c_w	0.1579196	0.2337851
c_{lf_w}	0.1402615	0.2222281
c_{sw}	0.07583085	0.08664242
c_{sr}	19811.93	1259.133
$c_{lf_{sr}}$	0.153532	0.2087366
$c_{s_{sr}}$	0.2334856	0.3364668
p	0.8981731	0.05417459
c_d	0.0006367383	0.1729927
c_{bf}	0.002343255	0.09554372
c_{so}	0.003922369	0.008363242

Text S2. Estimating plastic emission to the environment

To quantify the transport and retention of plastic on land and in rivers, we estimate the mass of micro- and macroplastic that enters the environment as an input to the model. The following section outlines the most relevant sources of micro- and macroplastic to land. We account for one source of macroplastic, mismanaged macroplastic waste (MaPW),[6] and three sources of microplastic inputs. These sources include, mismanaged plastics in the form of microplastics (MiPW),[7] tire wear particles (TWP)[8] and waste water treatment plants (WWTPs).[9] The model framework accounts solely for mismanaged macroplastics entering the river system through transport over land (diffuse source), while microplastics can enter the river system either through transport over land (MiPW and TWP) or directly into the river system through WWTPs, also known as point sources.[10] While we acknowledge other sources of plastic (i.e., microplastics from sports fields,[11] direct dumping of macroplastic into rivers,[12]), we recognize that these plastic sources provide a thorough representation of plastic pollution that is generated across Europe. The total mass of plastic per source is shown in Table S2.

Table S3. The total mass of each plastic source for all modelled river basins in Europe.

Plastic input	Total (Mt year^{-1})
MaPW	2.44
MiPW	0.0244
TWP	0.927
WWTPs	0.157

Macroplastic source

The largest source of plastic to the environment across the 8671 river basins is mismanaged macroplastic waste (2.44 Mt year^{-1}), MaPW. The spatial distribution of MaPW is adopted from Lebreton and Andrady[6], which gives estimates for all mismanaged plastic waste (MPW) 2015 at a 30 × 30 arc-second resolution. We determine that MPW consists of micro- and macroplastics. By comparing papers that quantify the presence of both micro-

and macroplastic in the environment,[13–15] we assume 99% of MPW is MaPW and 1% of MPW is MiPW. We, therefore, rescaled MPW to match this proportion for MaPW. Results were resampled (upscaled) to a 3 × 3 arc-second resolution to align with the spatial resolution of our model.

Microplastic sources

The first, and smallest source of microplastics, is a fraction of the MPW,[6] MiPW (0.02 Mt year^{-1}). MiPW accounts for the intentionally manufactured (primary) microplastics and fragmented macroplastics (secondary). As done for MaPW, MPW was rescaled to account for the proportion of MiPW.

Second, as a distinct source, we estimate the entry of TWPs to land. We estimate that 1.06 (0.52 - 1.5) kg cap^{-1} year^{-1} of tire wear particles are released by European countries.[16] Around 73.2% of the tire wear particles that are released by cars are deposited on land, therefore, not entering WWTPs.[17] We assume, therefore, an average diffuse input of tire wear particles of 0.77 kg cap^{-1} year^{-1} on land. We use a population density distribution for the year 2015[18] to determine the total mass of tire wear particles for each grid cell. This results in a total of 0.927 Mt year^{-1}), making it the largest source of microplastic.

Third, we account for WWTPs as point sources for microplastics to the river system (tire wear particles, laundry fibres and personal care products).[9] van Wijnen et al.[9] estimates that a direct influx of 0.2955 kg cap^{-1} year^{-1} of microplastics enters the river from WWTPs for OECD (Organisation for Economic Co-operation and Development) countries. We calculate the direct influx of microplastics at a river cell from WWTPs by locating the discharge location and population served using HydroWASTE data[19] and microplastic estimates from van Wijnen et al.[9]. The total mass of microplastics entering the river system directly through WWTPs equates to 0.16 Mt year^{-1}. Despite possible spatial variability in the efficiency of WWTPs in microplastic removal, we use one estimation for simplicity.

While we recognise that there are also other sources of microplastic waste, for example, sewage sludge used in agriculture[20] and artificial sports grounds,[21] we determine that our

model provides the most holistic overview of input of microplastics for such large scale modelling. We build on work from Schmidt et al.[22], Siegfried et al.[23], Strokal et al.[24], who model MiPW and/or WWTPs, by additionally modelling the large input of microplastic to land, TWP.

Text S3. Calibration and Validation: Harmonised field data

The minimum and maximum of the extrapolated field measurements were used for the model calibration for each river basin. The yearly mass flux estimates for plastic is shown below in Table S4 and Table S5.

Table S4. Part 1. Extrapolated field macroplastic transport measurements. Rivers in the validation set are indicated using a *v*.

	Location		Plastic transport ($\log_{10}$ kg year^{-1})		
River	Lat	Lon	Min	Max	Source
Adour	43.50	-1.48	1.99	3.82	González-Fernández et al.[25]
Arno	43.72	10.40	1.22	2.36	González-Fernández et al.[25]
Ave (v)	41.36	-8.73	1.73	2.79	González-Fernández et al.[25]
Besos	41.42	2.23	2.05	3.13	González-Fernández et al.[25]
Bojana (v)	42.05	19.49	1.94	3.24	González-Fernández et al.[25]
Chorokhi (v)	41.60	41.60	2.10	3.22	González-Fernández et al.[25]
Corrib	53.27	-9.06	1.57	2.99	González-Fernández et al.[25]
Danube (v)	45.40	29.58	2.60	3.90	González-Fernández et al.[25]
Douro	41.14	-8.60	1.95	3.14	González-Fernández et al.[25]
Drini (v)	19.64	41.78	2.60	3.74	González-Fernández et al.[25]
Ems	53.32	7.31	2.14	3.21	Schöneich-Argent et al.[26]
Guadalete (v)	36.60	-6.22	1.28	2.51	González-Fernández et al.[25]
Guadalhorce	36.68	-4.47	2.19	3.43	González-Fernández et al.[25]
Guadiana (v)	37.40	-7.44	2.30	3.40	González-Fernández et al.[25]
Hooksieler Binnentief	53.64	8.09	1.27	2.93	González-Fernández et al.[25]
Huveane	43.27	5.40	2.85	3.57	Tramoy et al.[27]
Ijssel	52.56	5.92	3.20	4.35	van Emmerik et al.[28]
Lamas	36.56	34.25	1.04	2.35	González-Fernández et al.[25]
Leca (v)	41.21	-8.61	1.35	2.75	González-Fernández et al.[25]
Lis (v)	39.88	-8.96	0.73	2.06	González-Fernández et al.[25]
Llobregat	41.32	2.11	2.05	3.50	González-Fernández et al.[25]
Magra	44.06	9.97	2.09	3.27	González-Fernández et al.[25]
Meuse	51.72	4.64	3.20	4.50	van Emmerik et al.[28]
Mondego	40.14	-8.71	2.14	3.52	González-Fernández et al.[25]

Table S4. Part 2. Extrapolated field macroplastic transport measurements. Rivers in the validation set are indicated using a *v*.

	Location		Plastic transport ($\log_{10}$ kg year^{-1})		
Natanebi	41.91	41.77	2.23	3.37	González-Fernández et al.[25]
Prego	41.03	-8.65	1.21	2.98	González-Fernández et al.[25]
Rhine	51.91	4.49	3.31	4.53	van Emmerik et al.[28]
Rhône (v)	43.68	4.62	2.26	3.48	González-Fernández et al.[25]
Rioni	42.20	41.66	1.82	3.21	González-Fernández et al.[25]
Seine	49.44	1.08	2.99	4.69	van Emmerik et al.[29]
Supsa (v)	42.02	41.76	2.13	3.65	González-Fernández et al.[25]
Têt	42.71	2.98	1.64	2.97	González-Fernández et al.[25]
Tiber	41.75	12.28	2.08	3.57	Cesarini et al.[30]
Valaderes	41.09	-8.66	1.34	2.48	González-Fernández et al.[25]
Vistula	54.26	18.95	2.32	3.85	González-Fernández et al.[25]
Weser	53.54	8.57	2.29	3.80	Schöneich-Argent et al.[26]

Table S5. Extrapolated field measurement for microplastic transport. Rivers in the validation set are indicated using a v.

River	Location		Plastic transport ($\log_{10}$ kg year^{-1})		Source
	Lat	Lon	Min	Max	
Anta (v)	40.76	-8.56	-0.08	2.91	Rodrigues et al.[31]
Arno (v)	43.71	10.37	3.04	6.03	Monnanni et al.[32]
Czarna Hańcza	54.08	22.98	1.85	4.84	Pol et al.[33]
Dambovita	44.40	26.23	5.43	8.43	Maria et al.[34]
Ebro	40.72	0.84	-0.22	2.77	Simon-Sánchez et al.[35]
Elbe	53.54	9.97	-0.93	2.06	Piehl et al.[36]
Ergene	41.22	27.49	2.18	5.17	Akdogan et al.[37]
Garonne	43.71	1.36	-0.54	3.09	de Carvalho et al.[38]
Henares	40.46	-3.42	-0.34	2.66	Schell et al.[39]
Krka	43.73	15.90	-0.65	2.88	Parać et al.[40]
Leen	53.01	-1.19	-0.69	2.35	Stanton et al.[41]
Liane	50.72	1.6	-0.43	3.20	Pasquier et al.[42]
Lis	39.88	-8.96	-0.39	4.20	Sá et al.[43]
Meuse	51.73	4.88	2.73	5.72	Mintenig et al.[44]
Mignone	42.18	11.74	2.19	5.17	Gallitelli et al.[45]
Po	44.95	12.44	2.42	5.40	Piehl et al.[36]
Rhine (v)	52.00	5.08	5.95	8.94	Mughini-Gras et al.[46]
Rhône	43.68	4.62	-1.45	1.53	Constant et al.[47]
Scheldt	51.23	4.40	1.25	6.46	Liu et al.[48]
Seine (v)	49.00	1.91	2.02	5.06	Treilles et al.[49]
Soar	52.87	-1.27	0.29	3.60	Stanton et al.[41]
Tame	53.42	-2.16	1.98	4.96	Woodward et al.[50]
Têt	42.71	2.98	0.18	3.16	Constant et al.[47]
Thames	51.51	0.12	2.75	5.74	Devereux et al.[51]
Trave (v)	53.95	10.86	-1.33	1.65	Piehl et al.[36]
Trent (v)	52.96	-2.20	-0.13	3.04	Stanton et al.[41]
Vipava	45.89	13.57	-2.11	0.87	Bertoli et al.[52]
Vistula (v)	53.18	23.10	1.37	4.36	Pol et al.[33]
Weser (v)	53.33	8.9	1.54	4.53	Roscher et al.[53]

Table S6. Results from the OAT analysis used to calculate uncertainty of macroplastic export. Results are given for six river basins for the original macroplastic export estimate, P10 and P90 (kg year^{-1}). All estimates are given to 1 decimal place.

Basin ID	Modelled output	P10	P90	- (%)	+ (%)
6303	97.4	47.2	292.4	5	200
7753	61551.4	20351.4	334485.6	67	443
7762	38717.9	15374.9	169513.9	60	338
9577	3.9	2.8	6.5	29	67
10734	95.2	36.7	398.5	61	319
10811	45552.6	16214.4	186749.8	64	310
			**Average**	**56**	**279**

Table S7. Results from the OAT analysis used to calculate uncertainty of microplastic export. Results are given for six river basins for the original microplastic export estimate, P10 and P90 (kg year^{-1}). All estimates are given to 1 decimal place.

Basin ID	Modelled output	P10	P90	- (%)	+ (%)
6303	12.5	11.6	13.5	7	8
7753	78.6	73.8	83.7	6	6
7762	55.3	51.9	58.6	6	6
9577	3.7	3.2	4.2	11	12
10734	0.5	0.5	0.6	6	7
10811	37.9	35.5	40.3	6	6
			**Average**	**7**	**8**

Table S8. Absolute percentile classes, used in Figure 5 in the main text.

	Export (t year^{-1})		Retention (t year^{-1})	
Percentile class	Macroplastic	Microplastic	Macroplastic	Microplastic
$< 70^{th}$	< 0.03	< 0.01	< 0.2	< 0.5
70 - 75th	0.03 - 0.05	0.01 - 0.02	0.2 - 0.3	0.5 - 0.8
75 - 80th	0.05 - 0.07	0.02 - 0.04	0.3 - 0.5	0.8 - 1.5
80 - 85th	0.07 - 0.1	0.04 - 0.09	0.5 - 0.9	1.5 - 3.0
85 - 90th	0.1 - 0.2	0.09 - 0.4	0.9 - 2.1	3.0 - 6.7
90 - 94th	0.2 - 0.6	0.4 - 2.1	2.1 - 5.6	6.7 - 15.4
94 - 96th	0.6 - 1.2	2.1 - 5.5	5.6 - 11.0	15.5 - 28.5
96 - 98th	1.2 - 3.4	5.5 - 17.2	11.0 - 34.3	28.5 - 65.3
98 - 99th	3.4 - 7.0	17.2 - 34.0	34.3 - 92.4	65.3 - 137.7
$> 99^{th}$	7.0 - 255.2	34.0 - 727.2	92.4 - 22875.9	137.7 - 22741.8

Table S9. Per capita percentile classes. Percentile colours correspond to those used in Figure 6 in the main text.

	Export (kg cap^{-1} year^{-1})		Retention (kg cap^{-1} year^{-1})	
Percentile class	Macroplastic	Microplastic	Macroplastic	Microplastic
$< 70^{th}$	< 0.8	< 0.6	< 2.2	< 8.9
70 - 75th	0.8 - 1.1	0.6 - 0.9	2.2 - 2.8	8.9 - 10.2
75 - 80th	1.1 - 1.7	0.9 - 1.3	2.8 - 3.7	10.2 - 11.9
80 - 85th	1.7 - 2.6	1.3 - 2.0	3.7 - 5.4	11.9 - 14.3
85 - 90th	2.6 - 4.5	2.0 - 3.3	5.4 - 11.4	14.3 - 19.9
90 - 94th	4.5 - 8.7	3.3 - 6.4	11.4 - 32.9	19.9 - 33.3
94 - 96th	8.7 - 13.9	6.4 - 11.1	32.9 - 53.3	33.3 - 60.5
96 - 98th	13.9 - 30.1	11.1 - 28.0	53.3 - 95.7	60.5 - 141.6
98 - 99th	30.1 - 50.5	28.0 - 68.6	95.7 - 151.2	141.6 - 283.2
$> 99^{th}$	50.5 - 778.3	68.6 - 2060.9	151.2 - 1771.4	283.2 - 14044.7

Table S10. Results from the Spearman's rank coefficient tests, between micro- and macroplastic export and retention ranks. We report Spearman's rho estimates, in addition to p-values.

Variable A	Variable B	p-value	rho
Macroplastic export rank	Microplastic export rank	<0.001	0.517
Macroplastic export rank	Macroplastic river retention rank	<0.001	0.793
Macroplastic export rank	Microplastic river retention rank	<0.001	0.590
Microplastic export rank	Macroplastic river retention rank	<0.001	0.478
Microplastic export rank	Microplastic river retention rank	<0.001	0.710
Macroplastic river retention rank	Microplastic river retention rank	<0.001	0.777

References

(1) Lehner, B.; Verdin, K.; Jarvis, A. New global hydrography derived from spaceborne elevation data. *Eos, Transactions, American Geophysical Union* **2008**, *89*, 93–94.

(2) Lehner, B.; Grill, G. Global river hydrography and network routing: baseline data and new approaches to study the world's large river systems. *Hydrological Processes* **2013**, *27*, 2171–2186, Data available at `https://www.hydrosheds.org`.

(3) Büttner, G.; Feranec, J.; Jaffrain, G.; Mari, L.; Maucha, G.; Soukup, T. The CORINE land cover 2000 project. *EARSeL eProceedings* **2004**, *3*, 331–346.

(4) European Commission, J. R. C. J. Global Land Cover 2000 database. 2003; `https://forobs.jrc.ec.europa.eu/glc2000`.

(5) Dörenkämper, M.; Olsen, B. T.; Witha, B.; Hahmann, A. N.; Davis, N. N.; Barcons, J.; Ezber, Y.; García-Bustamante, E.; González-Rouco, J. F.; Navarro, J.; others The making of the new european wind atlas–part 2: Production and evaluation. *Geoscientific Model Development Discussions* **2020**, *2020*, 1–37.

(6) Lebreton, L.; Andrady, A. Future scenarios of global plastic waste generation and disposal. *Palgrave Communications* **2019**, *5*, 1–11.

(7) Li, W.; Zu, B.; Yang, Q.; Guo, J.; Li, J. Sources, distribution, and environmental effects of microplastics: a systematic review. *RSC advances* **2023**, *13*, 15566–15574.

(8) Järlskog, I.; Jaramillo-Vogel, D.; Rausch, J.; Gustafsson, M.; Strömvall, A.-M.; Andersson-Sköld, Y. Concentrations of tire wear microplastics and other traffic-derived non-exhaust particles in the road environment. *Environment international* **2022**, *170*, 107618.

(9) van Wijnen, J.; Ragas, A. M.; Kroeze, C. Modelling global river export of microplastics

to the marine environment: Sources and future trends. *Science of the Total Environment* **2019**, *673*, 392–401.

(10) Strokal, M.; Spanier, J. E.; Kroeze, C.; Koelmans, A. A.; Flörke, M.; Franssen, W.; Hofstra, N.; Langan, S.; Tang, T.; van Vliet, M. T.; others Global multi-pollutant modelling of water quality: scientific challenges and future directions. *Current opinion in environmental sustainability* **2019**, *36*, 116–125.

(11) An, L.; Liu, Q.; Deng, Y.; Wu, W.; Gao, Y.; Ling, W. *Microplastics in terrestrial environments: Emerging contaminants and major challenges*; Springer, 2020; pp 143–159.

(12) Bruge, A.; Barreau, C.; Carlot, J.; Collin, H.; Moreno, C.; Maison, P. Monitoring litter inputs from the Adour River (Southwest France) to the marine environment. *Journal of Marine Science and Engineering* **2018**, *6*, 24.

(13) Garcés-Ordóñez, O.; Castillo-Olaya, V.; Espinosa-Díaz, L. F.; Canals, M. Seasonal variation in plastic litter pollution in mangroves from two remote tropical estuaries of the Colombian Pacific. *Marine Pollution Bulletin* **2023**, *193*, 115210.

(14) Haberstroh, C. J.; Arias, M. E.; Yin, Z.; Wang, M. C. Effects of urban hydrology on plastic transport in a subtropical river. *Acs Es&T Water* **2021**, *1*, 1714–1727.

(15) Schrank, I.; Löder, M. G.; Imhof, H. K.; Moses, S. R.; Heß, M.; Schwaiger, J.; Laforsch, C. Riverine microplastic contamination in southwest Germany: A large-scale survey. *Frontiers in Earth Science* **2022**, *10*, 794250.

(16) Kole, P. J.; Löhr, A. J.; van Belleghem, F. G.; Ragas, A. M. Wear and tear of tyres: a stealthy source of microplastics in the environment. *International journal of environmental research and public health* **2017**, *14*, 1265.

(17) Baensch-Baltruschat, B.; Kocher, B.; Kochleus, C.; Stock, F.; Reifferscheid, G. Tyre and road wear particles-A calculation of generation, transport and release to water and soil with special regard to German roads. *Science of the Total Environment* **2021**, *752*, 141939.

(18) Bondarenko, M.; others Individual Countries 1km Population Density (2000-2020). **2020**,

(19) Ehalt Macedo, H.; Lehner, B.; Nicell, J.; Grill, G.; Li, J.; Limtong, A.; Shakya, R. Distribution and characteristics of wastewater treatment plants within the global river network. *Earth System Science Data* **2022**, *14*, 559–577.

(20) Rolsky, C.; Kelkar, V.; Driver, E.; Halden, R. U. Municipal sewage sludge as a source of microplastics in the environment. *Current Opinion in Environmental Science & Health* **2020**, *14*, 16–22.

(21) Harusato, A.; Kato, M. Mechanisms of Generation and Ecological Impacts of Nano-and Microplastics from Artificial Turf Systems in Sports Facilities. *Environments* **2025**, *12*, 109.

(22) Schmidt, C.; Krauth, T.; Wagner, S. Export of plastic debris by rivers into the sea. *Environmental science & technology* **2017**, *51*, 12246–12253.

(23) Siegfried, M.; Koelmans, A. A.; Besseling, E.; Kroeze, C. Export of microplastics from land to sea. A modelling approach. *Water research* **2017**, *127*, 249–257.

(24) Strokal, M.; Vriend, P.; Bak, M. P.; Kroeze, C.; van Wijnen, J.; van Emmerik, T. River export of macro-and microplastics to seas by sources worldwide. *Nature Communications* **2023**, *14*, 4842.

(25) González-Fernández, D.; Cózar, A.; Hanke, G.; Viejo, J.; Morales-Caselles, C.;

Bakiu, R.; Barceló, D.; Bessa, F.; Bruge, A.; Cabrera, M.; others Floating macrolitter leaked from Europe into the ocean. *Nature Sustainability* **2021**, *4*, 474–483.

(26) Schöneich-Argent, R. I.; Dau, K.; Freund, H. Wasting the North Sea?–A field-based assessment of anthropogenic macrolitter loads and emission rates of three German tributaries. *Environmental Pollution* **2020**, *263*, 114367.

(27) Tramoy, R.; Blin, E.; Poitou, I.; Noûs, C.; Tassin, B.; Gasperi, J. Riverine litter in a small urban river in Marseille, France: Plastic load and management challenges. *Waste Management* **2022**, *140*, 154–163.

(28) van Emmerik, T.; De Lange, S.; Frings, R.; Schreyers, L.; Aalderink, H.; Leusink, J.; Begemann, F.; Hamers, E.; Hauk, R.; Janssens, N.; others Hydrology as a driver of floating river plastic transport. *Earth's Future* **2022**, *10*, e2022EF002811.

(29) van Emmerik, T.; Tramoy, R.; van Calcar, C.; Alligant, S.; Treilles, R.; Tassin, B.; Gasperi, J. Seine plastic debris transport tenfolded during increased river discharge. *Frontiers in Marine Science* **2019**, *6*, 642.

(30) Cesarini, G.; Crosti, R.; Secco, S.; Gallitelli, L.; Scalici, M. From city to sea: Spatiotemporal dynamics of floating macrolitter in the Tiber River. *Science of The Total Environment* **2023**, *857*, 159713.

(31) Rodrigues, M.; Abrantes, N.; Gonçalves, F.; Nogueira, H.; Marques, J.; Gonçalves, A. Spatial and temporal distribution of microplastics in water and sediments of a freshwater system (Antuã River, Portugal). *Science of the total environment* **2018**, *633*, 1549–1559.

(32) Monnanni, A.; Rimondi, V.; Morelli, G.; Nannoni, A.; Cincinelli, A.; Martellini, T.; Chelazzi, D.; Laurati, M.; Sforzi, L.; Ciani, F.; others Microplastics and microfibers contamination in the Arno River (Central Italy): Impact from urban areas and contribution to the Mediterranean Sea. *Science of the Total Environment* **2024**, *955*, 177113.

(33) Pol, W.; Żmijewska, A.; Stasińska, E.; Zieliński, P. Spatial–temporal distribution of microplastics in lowland rivers flowing through two cities (Ne Poland). *Water, Air, & Soil Pollution* **2022**, *233*, 140.

(34) Maria, C.; Deák, G.; Tudor, G.; Holban, E.; Zamfir, C.; Ivanov, A. A.; Grigore, G.; Rahim, N. Investigation of microplastics presence in the dambovita river. *International Journal of Conservation Science* **2023**, *14*, 663–670.

(35) Simon-Sánchez, L.; Grelaud, M.; Garcia-Orellana, J.; Ziveri, P. River Deltas as hotspots of microplastic accumulation: The case study of the Ebro River (NW Mediterranean). *Science of the total environment* **2019**, *687*, 1186–1196.

(36) Piehl, S.; Atwood, E. C.; Bochow, M.; Imhof, H. K.; Franke, J.; Siegert, F.; Laforsch, C. Can water constituents be used as proxy to map microplastic dispersal within transitional and coastal waters? *Frontiers in Environmental Science* **2020**, *8*, 92.

(37) Akdogan, Z.; Guven, B.; Kideys, A. E. Microplastic distribution in the surface water and sediment of the Ergene River. *Environmental Research* **2023**, *234*, 116500.

(38) de Carvalho, A. R.; Riem-Galliano, L.; Ter Halle, A.; Cucherousset, J. Interactive effect of urbanization and flood in modulating microplastic pollution in rivers. *Environmental Pollution* **2022**, *309*, 119760.

(39) Schell, T.; Hurley, R.; Nizzetto, L.; Rico, A.; Vighi, M. Spatio-temporal distribution of microplastics in a Mediterranean river catchment: the importance of wastewater as an environmental pathway. *Journal of hazardous materials* **2021**, *420*, 126481.

(40) Parać, M.; Cuculić, V.; Cukrov, N.; Geček, S.; Lovrić, M.; Cukrov, N. Microplastic distribution through the salinity gradient in a stratified estuary. *Water* **2022**, *14*, 3255.

(41) Stanton, T.; Johnson, M.; Nathanail, P.; MacNaughtan, W.; Gomes, R. L. Freshwater

microplastic concentrations vary through both space and time. *Environmental Pollution* **2020**, *263*, 114481.

(42) Pasquier, G.; Doyen, P.; Dehaut, A.; Veillet, G.; Duflos, G.; Amara, R. Vertical distribution of microplastics in a river water column using an innovative sampling method. *Environmental monitoring and assessment* **2023**, *195*, 1302.

(43) Sá, B.; Pais, J.; Antunes, J.; Pequeno, J.; Pires, A.; Sobral, P. Seasonal abundance and distribution patterns of microplastics in the Lis River, Portugal. *Sustainability* **2022**, *14*, 2255.

(44) Mintenig, S.; Kooi, M.; Erich, M.; Primpke, S.; Redondo-Hasselerharm, P.; Dekker, S.; Koelmans, A.; van Wezel, A. A systems approach to understand microplastic occurrence and variability in Dutch riverine surface waters. *Water research* **2020**, *176*, 115723.

(45) Gallitelli, L.; Cesarini, G.; Cera, A.; Sighicelli, M.; Lecce, F.; Menegoni, P.; Scalici, M. Transport and deposition of microplastics and mesoplastics along the river course: a case study of a small river in central Italy. *Hydrology* **2020**, *7*, 90.

(46) Mughini-Gras, L.; van der Plaats, R. Q.; van der Wielen, P. W.; Bauerlein, P. S.; de Roda Husman, A. M. Riverine microplastic and microbial community compositions: a field study in the Netherlands. *Water Research* **2021**, *192*, 116852.

(47) Constant, M.; Ludwig, W.; Kerhervé, P.; Sola, J.; Charrière, B.; Sanchez-Vidal, A.; Canals, M.; Heussner, S. Microplastic fluxes in a large and a small Mediterranean river catchments: The Têt and the Rhône, Northwestern Mediterranean Sea. *Science of the Total Environment* **2020**, *716*, 136984.

(48) Liu, Z.; Huang, Q.; Wang, H.; Zhang, S. An enhanced risk assessment framework for microplastics occurring in the Westerscheldt estuary. *Science of the Total Environment* **2022**, *817*, 153006.

(49) Treilles, R.; Gasperi, J.; Tramoy, R.; Dris, R.; Gallard, A.; Partibane, C.; Tassin, B. Microplastic and microfiber fluxes in the Seine River: flood events versus dry periods. *Science of the Total Environment* **2022**, *805*, 150123.

(50) Woodward, J.; Li, J.; Rothwell, J.; Hurley, R. Acute riverine microplastic contamination due to avoidable releases of untreated wastewater. *Nature Sustainability* **2021**, *4*, 793–802.

(51) Devereux, R.; Ayati, B.; Westhead, E. K.; Jayaratne, R.; Newport, D. "The great source" microplastic abundance and characteristics along the river Thames. *Marine pollution bulletin* **2023**, *191*, 114965.

(52) Bertoli, M.; Lesa, D.; Pastorino, P.; Mele, A.; Anselmi, S.; Barceló, D.; Prearo, M.; Renzi, M.; Pizzul, E. Microplastic patterns in riverine waters and leaf litter: Leaf bag technique to investigate the microplastic accumulation trends in lotic ecosystems. *Journal of Contaminant Hydrology* **2023**, *259*, 104253.

(53) Roscher, L.; Fehres, A.; Reisel, L.; Halbach, M.; Scholz-Böttcher, B.; Gerriets, M.; Badewien, T. H.; Shiravani, G.; Wurpts, A.; Primpke, S.; others Microplastic pollution in the Weser estuary and the German North Sea. *Environmental Pollution* **2021**, *288*, 117681.
